# Diagnostic Challenge of a Vascular Liver Tumor With Pulmonary Hemorrhagic Metastases: A Case Report

**DOI:** 10.7759/cureus.105700

**Published:** 2026-03-23

**Authors:** Hana N P, Afrah Fathima Karimbanakkal Edakkattu

**Affiliations:** 1 Department of Radiology, Kunhitharuvai Memorial Charitable Trust (KMCT) Medical College, Kozhikode, IND; 2 Medical School, Government Medical College, Manjeri, Kerala University of Health Sciences, Manjeri, IND

**Keywords:** case reports, chronic liver disease (cld), hemangiosarcoma, lung neoplasms, multiple pulmonary nodules

## Abstract

We report the case of a 64-year-old man with chronic liver disease who presented with abdominal discomfort, anorexia, and progressive weight loss, initially suspected to have hepatocellular carcinoma based on a large hepatic mass lacking definitive radiologic hallmarks. Multiphase CT demonstrated heterogeneous peripheral enhancement without classical washout, while thoracic imaging revealed numerous bilateral pulmonary nodules surrounded by ground-glass halos, indicating hemorrhagic metastases. These thoracic findings proved pivotal in directing diagnostic suspicion toward a vascular neoplasm. Ultrasound-guided liver biopsy confirmed high-grade hepatic angiosarcoma, with tumor cells showing ERG positivity and weak CD31 expression, and lacking hepatocellular or epithelial markers. The patient experienced rapid clinical deterioration with intrapulmonary hemorrhage, multiorgan dysfunction, and refractory shock, culminating in death shortly after diagnosis. This case highlights the diagnostic complexity of hepatic angiosarcoma and underscores the importance of integrating thoracic imaging with hepatic evaluation, particularly when liver findings are atypical.

## Introduction

Hepatic angiosarcoma is a rare and highly aggressive malignant tumor arising from vascular endothelial cells, accounting for fewer than 2% of primary hepatic malignancies [1]. Despite its rarity, it carries an extremely poor prognosis, with most patients presenting at advanced stages of disease and median survival often fewer than six months. Clinical manifestations are typically nonspecific, including abdominal discomfort, weight loss, and constitutional symptoms, which frequently lead to delayed diagnosis [2].

Radiologic evaluation of hepatic angiosarcoma can be challenging because imaging findings are heterogeneous and may mimic more common hepatic malignancies such as hepatocellular carcinoma (HCC) or intrahepatic cholangiocarcinoma [1,3].

In patients with underlying chronic liver disease, the diagnostic complexity is further amplified, as clinicians often initially suspect HCC. In addition, serum biomarkers such as alpha-fetoprotein usually lack diagnostic specificity in these cases [3].

Pulmonary metastases are common in hepatic angiosarcoma and may present as hemorrhagic nodules with a surrounding ground-glass halo on CT, referred to as the “CT halo sign.” Although this sign is not pathognomonic, it may serve as an important diagnostic clue suggesting an underlying vascular malignancy [1-3].

This case report highlights a diagnostically challenging case of primary hepatic angiosarcoma (PHA) in a patient with chronic liver disease, where thoracic CT findings played a crucial role in redirecting the differential diagnosis. The case emphasizes the importance of integrating thoracic and hepatic imaging findings when evaluating atypical hepatic masses.

## Case presentation

Patient information

The patient was a 64-year-old Indian male with significant past medical history, including type 2 diabetes mellitus, hypertension, and hypothyroidism. He had no known drug allergies. Baseline functional status was PS 2 [4], with recent deterioration attributed to constitutional symptoms. The patient initially presented with right-sided abdominal discomfort for two weeks, severe anorexia, substantial weight loss, and intermittent lower-limb edema. No history of gastrointestinal bleeding, jaundice, alcohol abuse, or hepatotoxic drug exposure was documented. Prior imaging from an outside facility revealed chronic liver disease with a large space-occupying lesion in the left hepatic lobe. He presented to the tertiary care center for further evaluation and tissue diagnosis. There was no documented family history of hepatic malignancy or genetic cancer syndromes (Table 1).

**Table 1:** Timeline of clinical events. CLD = chronic liver disease; SOL = space-occupying lesion; AFP = alpha-fetoprotein; LFTs = liver function tests; USG = ultrasonography; IHC = immunohistochemistry; ERG = ETS-related gene (erythroblast transformation-specific-related gene); HepPar-1 = hepatocyte paraffin 1 (hepatocyte-specific antigen); CK = cytokeratin; Na⁺ = serum sodium; ED = emergency department; POCUS = point-of-care ultrasound; IVC = inferior vena cava

Date	Event
Day 1	The patient develops right abdominal discomfort, anorexia, weight loss, and intermittent leg edema
Day 14	Shows CLD with regenerative nodules and a large left lobe SOL
Day 15	Outpatient evaluation performed at our center, including elevated glucose, normal AFP, baseline LFTs, and normal creatinine
Day 16	Prothrombin time studies performed—coagulation preserved
Day 17	USG-guided liver biopsy performed
Day 21	Histopathology suggests poorly differentiated malignant neoplasm, favoring angiosarcoma; IHC (CD31 weak+, ERG+, HepPar-1−, CK−) confirms hepatic angiosarcoma
Day 23	Scheduled follow-up with medical oncology (before clinical decline)
Day 24	The patient develops fever, dyspnea, and desaturation at home; biochemical derangements begin (Na⁺ = 129 mmol/L)
Day 24	Presents to the ED in acute respiratory failure; intubated; POCUS shows B-lines and collapsed IVC; labs show rising creatinine and profound hepatic injury
Day 25	Despite maximal resuscitation, the patient succumbs to refractory septic shock and intrapulmonary hemorrhage

Clinical assessment

Admission findings

On admission, the patient was afebrile with stable hemodynamics. Physical examination revealed a soft, non-tender abdomen and unremarkable cardiopulmonary findings. Point-of-care ultrasound (POCUS) (GE LOGIQ E9 (GE Healthcare, Wauwatosa, WI, USA)) demonstrated a collapsed inferior vena cava, suggesting hypovolemia, mild left ventricular dysfunction with regional wall motion abnormalities, extensive bilateral B-lines indicating interstitial involvement or alveolar hemorrhage, and there was no evidence of deep venous thrombosis or hydronephrosis.

Diagnostic assessment

Laboratory Investigations

Table 2 presents a summary of the patient’s laboratory findings.

**Table 2:** Summary of the patient’s laboratory findings. *: Control = 12.8 seconds; INR = 1.74. All laboratory parameters presented represent values at admission unless otherwise indicated. ↑ = elevated value; ↓ = decreased value

Laboratory test	Patient’s value	Normal range
Sodium (Na⁺)	129–130 mmol/L	135–145 mmol/L
Creatinine	1.9 mg/dL (↓)	0.6–1.2 mg/dL
Bilirubin (total)	3.4 mg/dL (↑)	0.1–1.2 mg/dL
Aspartate aminotransferase (AST)	94–563 U/L (↑)	10–40 U/L
Alanine aminotransferase	Elevated (higher than AST) (↑)	7–56 U/L
Alkaline phosphatase	227 U/L (↑)	44–147 U/L
Albumin	2.0 g/dL (↓)	3.5–5.0 g/dL
Hemoglobin (before biopsy)	9.4 g/dL (↓)	13.5–17.5 g/dL
Hemoglobin (after biopsy)	8.0 g/dL (↓)	13.5–17.5 g/dL
Prothrombin time (PT with international normalized ratio)*	20.1 seconds 1.74	12–16 seconds

Given the absence of coagulation abnormalities and lack of focal bleeding complications, the hemoglobin decline was more likely attributable to ongoing tumor-related hemorrhagic activity, which is consistent with the known biology of hepatic angiosarcoma and its propensity for intratumoral and systemic bleeding.

Imaging studies

Abdominal CT

**Figure 1:**
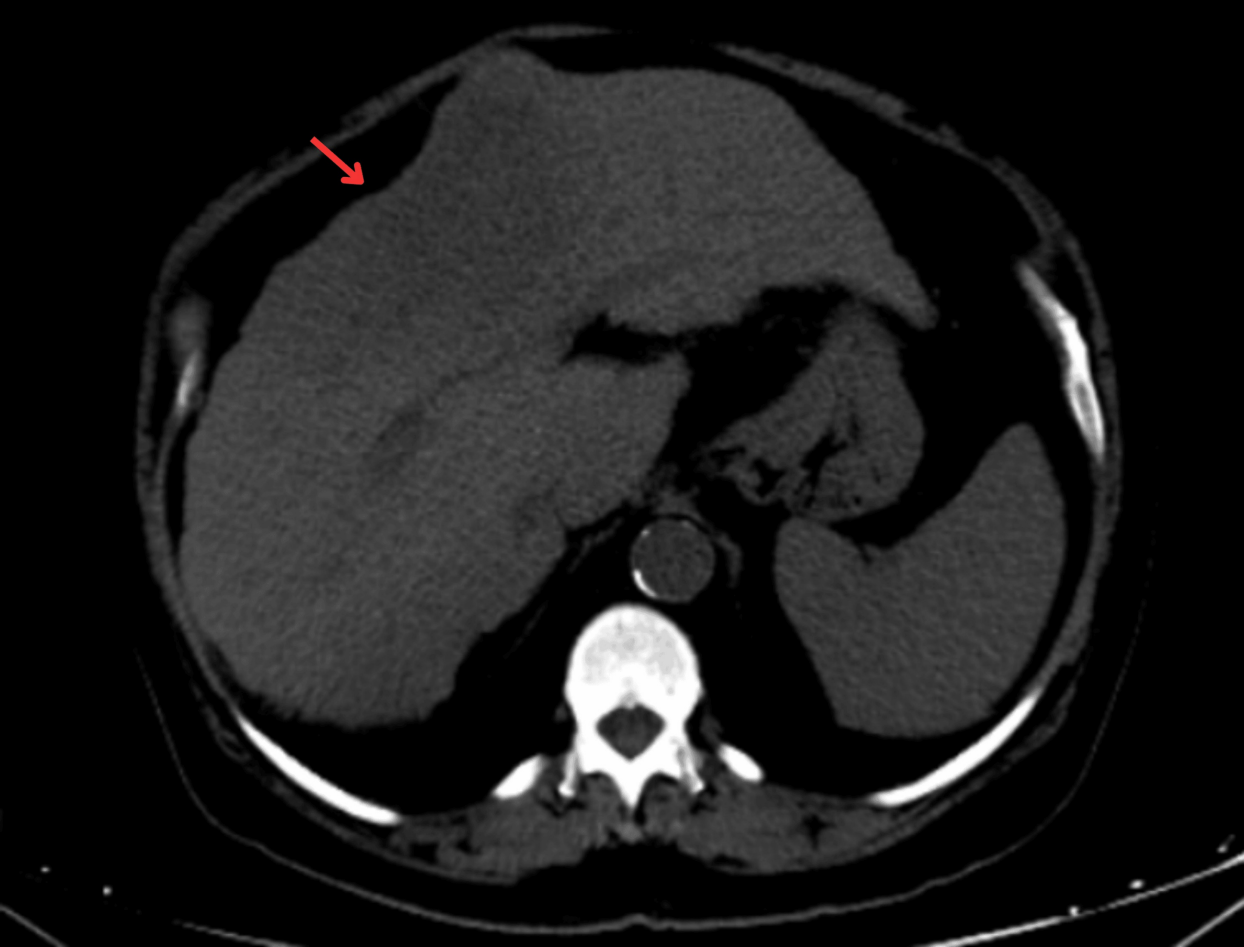
Axial non-contrast CT of the abdomen showing a large, ill-defined hypoattenuating mass occupying the left hepatic lobe, with heterogeneous internal density and loss of normal lobar architecture. The liver shows background features suggestive of chronic liver parenchymal disease, including a nodular surface, irregular borders, and left lobe hypertrophy. No intralesional calcification, macroscopic fat density, or hemorrhage is visible. Findings are consistent with a large infiltrative hepatic lesion, later confirmed as hepatic angiosarcoma.

**Figure 2:**
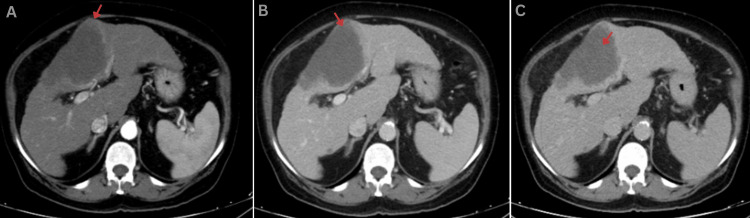
Axial contrast-enhanced CT of the abdomen. Panel A (arterial phase) demonstrates a large, lobulated, heterogeneously enhancing mass occupying segment IV of the liver, showing peripheral rim enhancement. Panel B (venous phase) shows persistent heterogeneous enhancement without washout, with poorly defined infiltrative margins. Panel C (delayed phase) reveals irregular progressive centripetal fill-in enhancement within the lesion.

**Figure 3:**
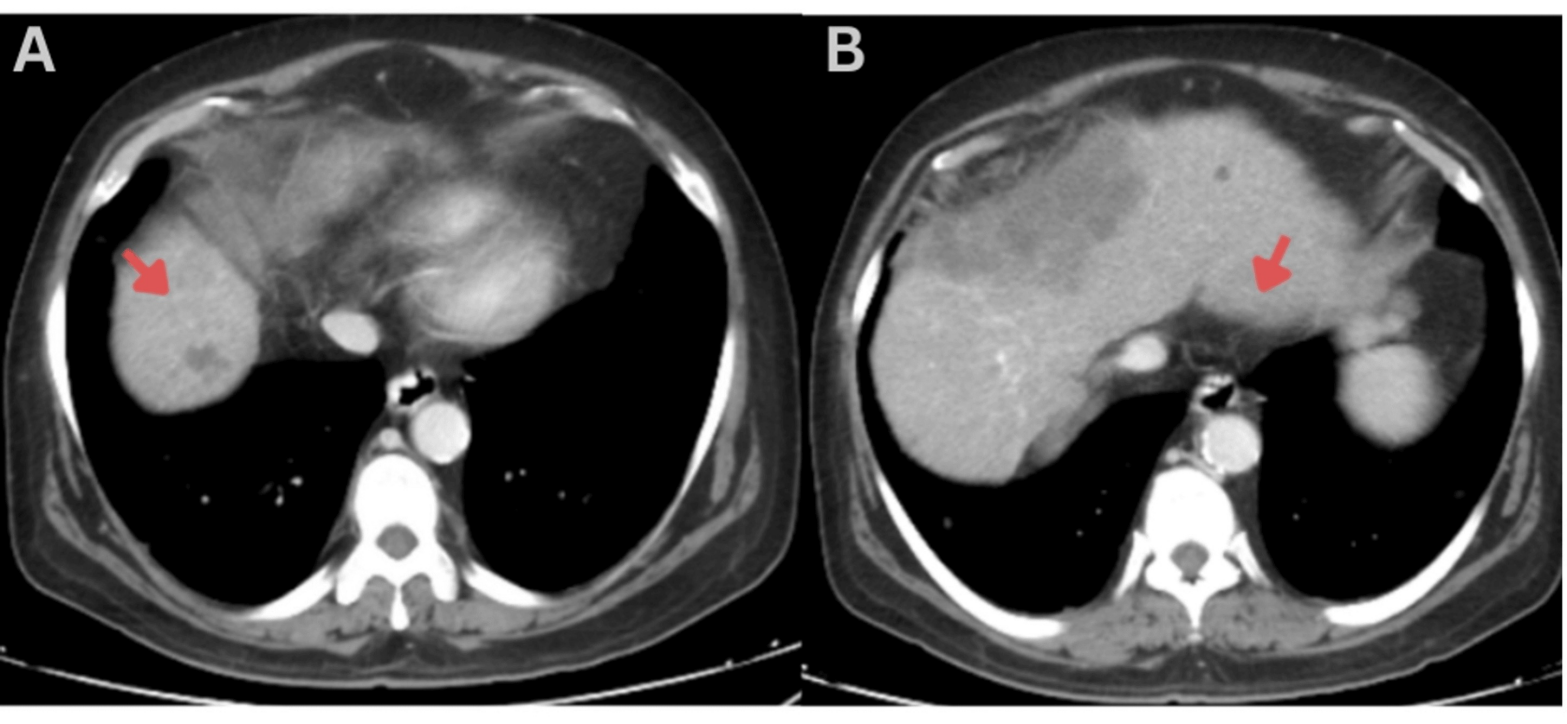
Axial contrast-enhanced CT of the abdomen demonstrating multiple similar hepatic satellite lesions (arrows) involving both lobes of the liver, with the largest lesion located in segment VII.

**Figure 4:**
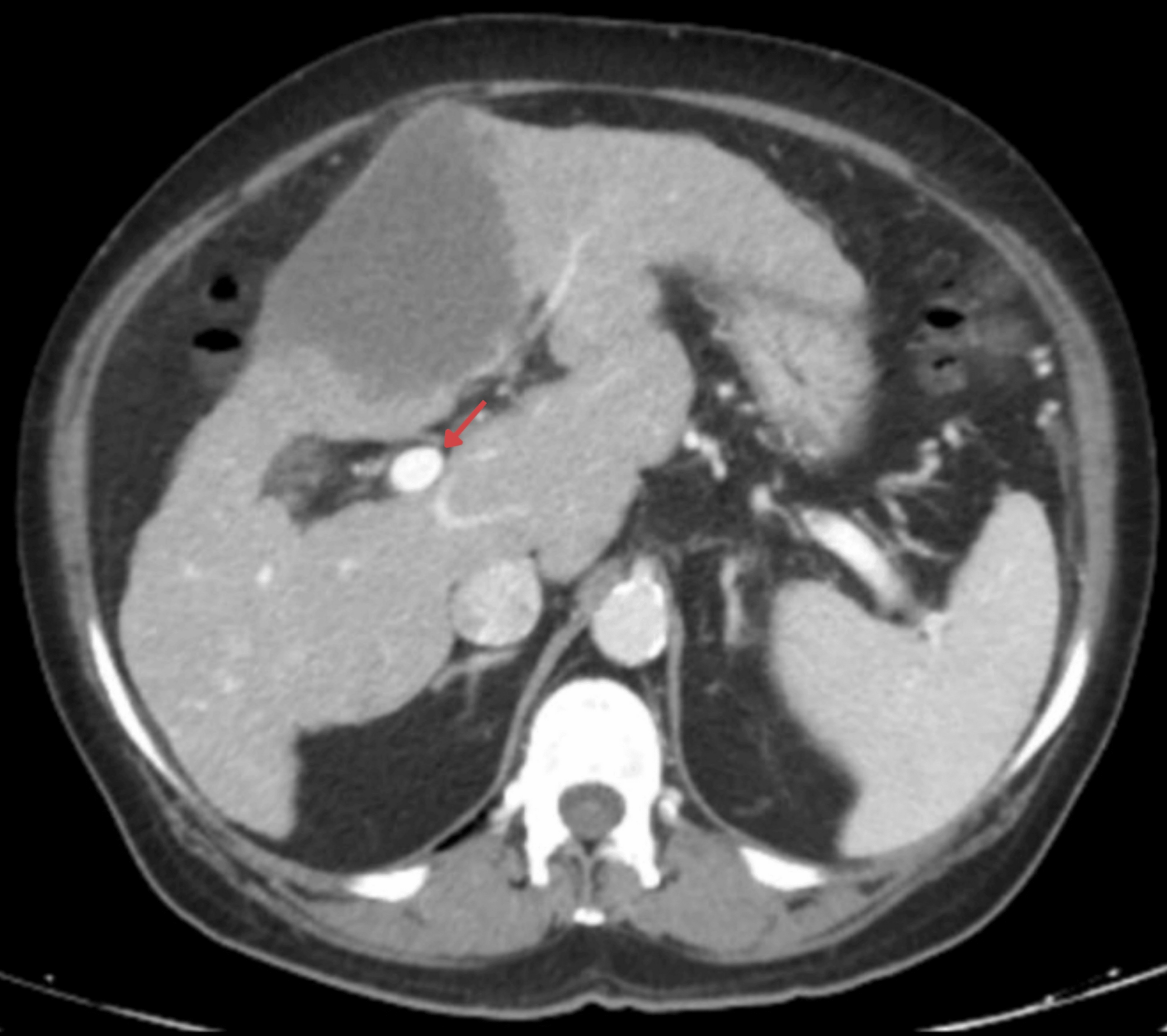
Axial contrast-enhanced CT of the abdomen (portal venous phase) demonstrating opacification of the portal vein (arrow), indicating absence of macrovascular invasion, a feature commonly seen in hepatocellular carcinoma and cholangiocarcinoma, thereby supporting the exclusion of these differentials.

Contrast-enhanced CT demonstrated a large, lobulated, heterogeneously enhancing mass in hepatic segment IV with peripheral rim enhancement (arterial phase), persistent heterogeneous enhancement without washout (venous phase), and irregular centripetal fill-in (delayed phase). No macrovascular invasion or lymphadenopathy was seen, findings which argued against typical HCC or cholangiocarcinoma (Figures 1-4).

Thoracic CT

**Figure 5:**
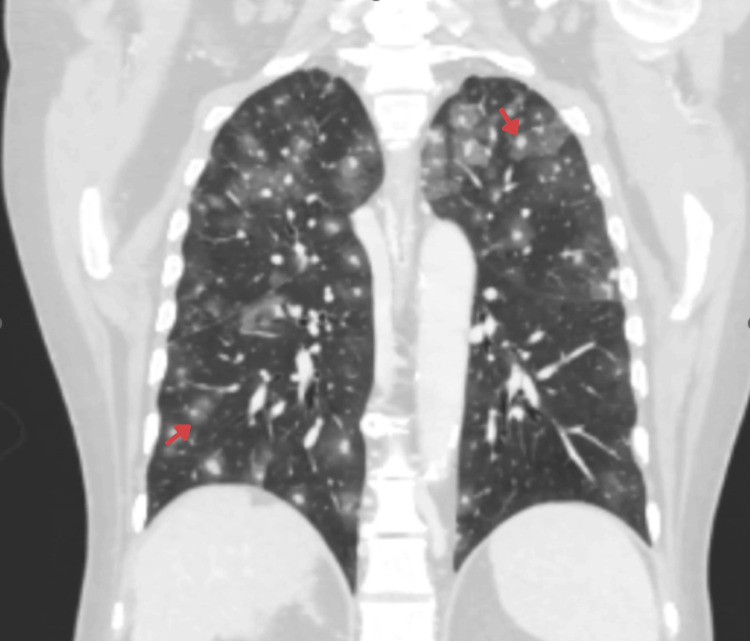
Coronal CT of the thorax showing numerous bilateral pulmonary nodules. Many nodules are surrounded by a peripheral ground-glass halo, the “halo sign,” indicating perilesional hemorrhage which is highly suggestive of hemorrhagic metastases from angiosarcoma.

Initial lower thoracic images demonstrated a few pulmonary nodules with peripheral ground-glass halos in the bilateral lower lobes. This prompted a complete thoracic CT evaluation, which revealed numerous bilateral pulmonary nodules ranging from 4 mm to 2 cm, each surrounded by a ground-glass halo, the “halo sign,” indicating perilesional hemorrhage (Figure 5). This imaging feature was decisive, strongly pointing toward hemorrhagic metastases from angiosarcoma and distinguishing them from metastases of HCC or cholangiocarcinoma.

Histopathology and immunohistochemistry

**Figure 6:**
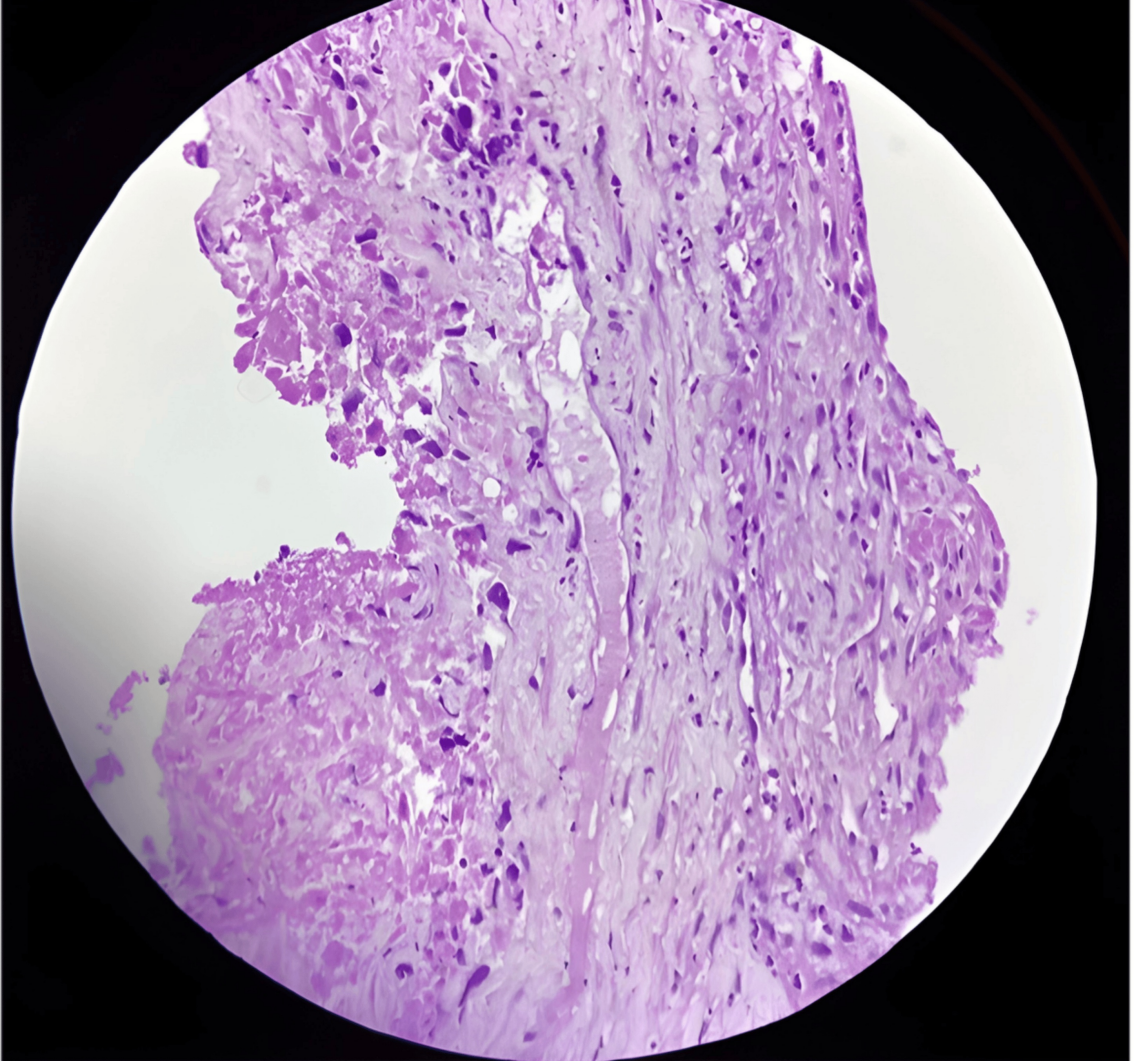
Hematoxylin and eosin-stained section from the ultrasound-guided liver biopsy demonstrates malignant spindle to epithelioid cells arranged in disorganized vascular channels, infiltrating the hepatic parenchyma. The tumor shows marked pleomorphism, hyperchromatic nuclei, prominent nucleoli, and areas of extensive necrosis. Scattered atypical endothelial cells line irregular, anastomosing vascular spaces, a characteristic feature of hepatic angiosarcoma.

Histopathological examination of the liver biopsy revealed a highly malignant vascular neoplasm composed of spindle-to-epithelioid cells arranged in irregular, anastomosing vascular channels infiltrating the hepatic parenchyma. The tumor demonstrated marked nuclear pleomorphism, hyperchromasia, prominent nucleoli, and extensive areas of necrosis, features consistent with a high-grade angiosarcoma. Immunohistochemical analysis further supported this diagnosis. The neoplastic endothelial cells showed strong nuclear positivity for ERG, a highly specific marker of vascular differentiation, and weak membranous positivity for CD31. In contrast, the tumor cells were negative for HepPar-1 and cytokeratin, effectively excluding HCC and cholangiocarcinoma. The combined histologic architecture and immunophenotypic profile confirmed the diagnosis of PHA (Figure 6).

Differential diagnosis

In the initial evaluation of a hepatic mass in a patient with underlying chronic liver disease, HCC was the foremost diagnostic consideration. However, the lesion lacked the classical imaging hallmarks of HCC, such as arterial-phase hyperenhancement with venous or delayed washout, and no macrovascular invasion was identified. Intrahepatic cholangiocarcinoma was also considered, but the absence of biliary ductal dilatation, lymphadenopathy, or a desmoplastic stromal pattern made this diagnosis less convincing. Metastatic carcinoma from an unknown primary constituted another important possibility, particularly given the multifocal appearance of the hepatic lesion, yet the immunohistochemical profile did not support an epithelial origin. A poorly differentiated sarcoma was entertained as well, though the specific endothelial immunophenotype ultimately argued against this broader category. The diagnostic shift occurred following thoracic CT imaging, which demonstrated numerous pulmonary nodules with characteristic ground-glass halos, findings strongly suggestive of hemorrhagic metastases from angiosarcoma. Together with the immunohistochemical pattern showing ERG positivity and absence of hepatocellular or cholangiocellular markers, these findings solidified hepatic angiosarcoma as the final diagnosis.

Treatment and clinical course

Management included packed red blood cell transfusion, supplemental oxygen, peri-procedural steroids and antihistamines, diabetes and thyroid optimization, and supportive medications such as ursodeoxycholic acid and proton pump inhibitors.

Terminal hospitalization

The patient presented in acute respiratory failure requiring mechanical ventilation (FiO₂ of 75%), triple inotropic support, broad-spectrum antibiotics, and aggressive fluid resuscitation. POCUS showed features consistent with intrapulmonary hemorrhage and shock physiology. Despite maximal therapy, he developed progressive metabolic acidosis, refractory hypotension, and multiorgan dysfunction, culminating in death within hours of admission.

## Discussion

PHA is an exceptionally rare and highly aggressive malignant vascular tumor arising from endothelial cells, accounting for approximately 2% of primary hepatic malignancies. The disease is associated with an extremely poor prognosis, with only a limited number of cases reported annually worldwide. Clinically, PHA typically presents with nonspecific symptoms such as abdominal discomfort, anorexia, weight loss, or fatigue, frequently resulting in delayed diagnosis and advanced-stage disease at presentation [5,6]. In the present case, the patient exhibited constitutional symptoms without distinctive biochemical abnormalities, including a normal alpha-fetoprotein level, a finding that has been consistently reported in previous studies and further complicates early diagnostic recognition [2,6].

Radiologic evaluation of PHA remains challenging because of its heterogeneous imaging characteristics. As described by Koyama et al., PHA may manifest radiologically as multiple nodules, a dominant mass, or a diffuse infiltrative lesion, with substantial variability on CT and MRI [7]. Imaging reviews have further demonstrated that PHA frequently exhibits peripheral arterial-phase enhancement with progressive centripetal fill-in, patterns that can mimic HCC or hypervascular cholangiocarcinoma, particularly in patients with underlying chronic liver disease [2]. This overlap has important clinical implications, as clinicians often initially attribute hepatic lesions in cirrhotic or chronically diseased livers to HCC. In our patient, multiphase CT demonstrated irregular peripheral enhancement with delayed progressive fill-in, a pattern consistent with vascular tumor architecture described in prior radiologic reports [1,8,9]. Nevertheless, imaging alone was insufficient to establish a definitive diagnosis, reflecting the well-recognized limitation of radiologic findings in differentiating PHA from other hepatic malignancies and reinforcing the need for histopathologic confirmation [9,10].

Histopathological examination remains the cornerstone for definitive diagnosis. In this case, microscopic evaluation revealed spindle-to-epithelioid malignant endothelial cells arranged in irregular, anastomosing vascular channels with marked nuclear pleomorphism, hyperchromasia, and areas of necrosis, features characteristic of hepatic angiosarcoma. Immunohistochemistry further supported this diagnosis, demonstrating ERG positivity and CD31 expression while lacking hepatocellular (HepPar-1) and epithelial (cytokeratin) markers. These findings are consistent with previously described immunophenotypic profiles of vascular tumors, where CD31 is considered one of the most reliable endothelial markers, and ERG represents a highly sensitive nuclear transcription factor indicating vascular differentiation [9,10]. The concordance between the pathological findings in our case and previously reported features supports the diagnostic accuracy despite the nonspecific radiologic presentation.

An important distinguishing feature of this case was the presence of multiple hemorrhagic pulmonary metastases demonstrating the CT “halo sign,” characterized by nodules surrounded by ground-glass opacities reflecting perilesional hemorrhage. Although the halo sign is not specific and may occur in infectious, inflammatory, or neoplastic conditions, it is a recognized radiologic manifestation of hemorrhagic metastases from vascular tumors [7]. In the context of an atypical hepatic mass, the presence of numerous pulmonary nodules with surrounding ground-glass halos provided a critical diagnostic clue suggesting a vascular malignancy rather than more common hepatic tumors such as HCC or cholangiocarcinoma. Previous studies have identified the lungs as one of the most frequent sites of extrahepatic metastasis in PHA [2-5]; however, hemorrhagic halo-type metastases remain relatively underrecognized in clinical practice. In our case, the thoracic CT findings played a pivotal role in redirecting the diagnostic consideration toward angiosarcoma, highlighting the importance of integrating thoracic imaging when evaluating atypical hepatic lesions.

Despite improvements in imaging and diagnostic techniques, PHA continues to be associated with extremely poor clinical outcomes. A 2015 study reported a median survival of approximately 1.9 months following diagnosis, significantly worse than that observed in HCC [5]. Surgical resection remains the only treatment associated with a meaningful survival benefit, with selected cases demonstrating median survival times ranging from 6 to 18 months [2,5]. However, most patients present with advanced or metastatic disease, and true surgical resectability is achieved in fewer than 20% of cases [2,5]. The rapid clinical deterioration observed in our patient, characterized by respiratory failure, shock, and multiorgan dysfunction within a short interval after diagnosis, is consistent with the aggressive biological behavior of metastatic PHA. Multifocal intrahepatic involvement and hemorrhagic pulmonary metastases, both present in this case, have been associated with particularly poor outcomes [1,2,5].

Therapeutic options for advanced PHA remain limited. Systemic chemotherapy has demonstrated only modest and transient benefits, with reported median survival durations of approximately five months in treated patients and no established regimen providing durable disease control [5]. Locoregional interventions such as transarterial chemoembolization or transarterial embolization may provide temporary disease stabilization in selected patients with localized disease but are generally ineffective in disseminated or rapidly progressive cases [10]. In our patient, the rapid progression of respiratory and systemic deterioration precluded the initiation of oncologic therapy, illustrating the real-world challenges associated with managing advanced PHA.

The present case adds incremental value to the existing literature in several respects. First, it highlights the diagnostic importance of thoracic CT findings in patients presenting with atypical hepatic masses. In this case, the identification of hemorrhagic pulmonary nodules with a halo sign provided an early clue suggesting a vascular malignancy and prompted reconsideration of the initial differential diagnosis. Second, the case illustrates the diagnostic difficulty of distinguishing hepatic angiosarcoma from HCC in patients with underlying chronic liver disease when imaging findings are atypical. Finally, the extremely rapid clinical deterioration due to hemorrhagic pulmonary metastases emphasizes the aggressive nature of metastatic PHA and underscores the importance of early tissue diagnosis when unusual imaging features are encountered.

Clinical takeaway

In patients with atypical hepatic masses, the presence of hemorrhagic pulmonary nodules with a CT halo sign should prompt strong consideration of a vascular malignancy such as hepatic angiosarcoma and early pursuit of tissue diagnosis.

## Conclusions

PHA remains a rare but highly aggressive vascular malignancy that frequently presents with nonspecific clinical and radiologic findings. In patients with atypical hepatic masses, particularly when imaging features are not characteristic of HCC, clinicians should consider the possibility of vascular tumors in the differential diagnosis. The presence of multiple pulmonary nodules with surrounding ground-glass halos on thoracic CT may represent hemorrhagic metastases and can provide an important radiologic clue suggesting angiosarcoma. This case highlights the importance of integrating thoracic imaging findings with hepatic evaluation and emphasizes the role of histopathologic confirmation in establishing the diagnosis. Although therapeutic options remain limited and prognosis is poor in advanced disease, early recognition of atypical imaging patterns may facilitate timely diagnosis and appropriate clinical decision-making.
